# Nursing: dialogue with the past in the commitment to the present

**DOI:** 10.1590/1518-8345.0000-3220

**Published:** 2019-10-07

**Authors:** Emilia Luigia Saparoti Angerami

**Affiliations:** 1 Universidade de São Paulo, Escola de Enfermagem de Ribeirão Preto, PAHO/WHO Collaborating Centre for Nursing Research Development, Ribeirão Preto, SP, Brazil.

**Figure:**
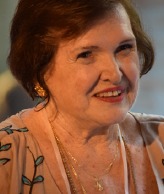

In the 1960s, the world went through profound political-social transformations; nursing, in this context, expanded spaces and assumed new tasks. The reduced number of nurses was asked to treat patients, administer wards and train human resources.

Nursing entered the university, assuming research as a promising element for professional development. The first few studies, of the descriptive type, were important for showing the *status quo* of the profession, the human capital available and to be trained in relation to the gaps in the services and in education, as well as to be researchers. The problems investigated answered the questions made, contributing to the advancement of knowledge and, above all, to awaken interest in the continuity of research and in the multiplication of the themes under study.

In this century, in 2019, the global Nursing Now campaign* of the World Health Organization (WHO), assumed by the international leaders of nursing, puts us in the central position to “reach the goal of universal coverage” of the populations, among other actions. Topics that have long been concerns in my professional life associated with teaching, practice and research in human resources remain present in the face of problems related to the low number of professionals in the services, unjust salaries, bad working conditions, lack of recognition of the work of nurses, difficulty in recruiting nurses and students, staff retention and brain drain. Additionally, gender issues, low wages, violence in the services, overload of the work of women, precariousness of human and material resources and poor working conditions have also been subjects of concern.

Two theses of professors from the University of São Paulo’s School of Nursing in Ribeirão Preto were milestones of research in nursing in Brazil and Latin America, and show the concern over the social recognition of the profession. The conclusions of the first thesis^(^ 1 ^)^ show the need for the community to become aware of the role of nursing; according to the authors, nurses, through their professional performance, should contribute so that this degree of perception is achieved quickly, suggesting that: “nurses are responsible for the construction of their own image”^(^ 1 ^)^. In the second thesis, the researcher, a sociologist, when analyzing nursing as a profession in a teaching hospital, reports the inconsistency and lack of definition of roles among the nursing categories, with the profession being poorly integrated into the social system of hospitals and undervalued in global society^(^ 2 ^)^. The topicality of these results reveals the value of replicating studies from time to time, as while the temporal comparison is valuable, it is possible to go beyond it, reviewing strategies, advances and setbacks, motivations and even making new questions involving persistent themes.

After forty years of Alma Ata^(^ 3 ^)^ and considering the profound transformation that took place in health care services with the assumption of the prevention of diseases as main goal, nursing was considered key to achieving the goals proposed by WHO, “Health for all by the year 2000”. The commitment of nurses to achieve this goal was intense, in the organization of services, in government programs and in the training of human resources. In Brazil and throughout Latin America, several programs have been created and their contribution to health remains vigorous.

The recent call to nurse leaders to once again assume a central position for the success of the new WHO proposal induces essential reflections, as the current working conditions are considered unsatisfactory and the human and material resources available are insufficient to meet the population that knocks on the doors of health care services and is denied shelter, which in turn creates problems of legal and ethical proportions.

Transforming services or changing paradigms presupposes the group’s adherence to the project and acceptance of the empowerment of leadership, not as an attribute imposed on the individual, although its development is feasible. Being a leader implies changes in power relations because it is a process grounded in human relations.

In a world in constant transformation, where technology extinguishes and creates professions as if by magic, paradigms are built and deconstructed at the speed of light, making people tense and competitive in the pursuit of purposes, and sometimes incapable of dealing with small slips.

In a constantly moving environment, nurses must be well informed and ready to present innovative proposals, maintaining an ethical stance in view of the responsibility assumed by them in the exercise of their profession. In the position of leaders, they are those in charge of the management of situations and with the competence to reduce frictions that can cause uncontrollable crises; the ability of the leader manifests itself especially in conflicts.

The respect for the work performed begins at the base of the pyramid, as it is in the you/me relationship, with the recognition of the other as an equal, that the first movement of leadership is revealed. If patients do not know the nurses treating them, they will not know when and how to count on these nurses and their responsibility for the quality of care.

Being a leader is a compromise, one that nurses are being asked to assume once more at this time.

## References

[1] Alcantara G de (1963). Enfermagem moderna como categoria profissional: obstáculos a sua expansão na sociedade brasileira.

[2] Ferreira-Santos C (1968). A enfermeira como categoria ocupacional num moderno hospital-escola brasileiro.

[3] Organização Pan-americana de Saúde, Organização Mundial de Saúde (1978). Declaração de Alma-Ata.

